# An Overview of Curcumin in Neurological Disorders

**DOI:** 10.4103/0250-474X.65012

**Published:** 2010

**Authors:** S. K. Kulkarni, A. Dhir

**Affiliations:** Pharmacology Division, University Institute of Pharmaceutical Sciences, Panjab University, Chandigarh-160014, India; 1 Department of Neurobiology, University of California Davis Medical Center, Sacramento California, 95817, USA

**Keywords:** Curcumin, diabetic neuropathy, major depression, tardive dyskinesia

## Abstract

Curcumin, the principal curcuminoid found in spice turmeric, has recently been studied for its active role in the treatment of various central nervous system disorders. Curcumin demonstrates neuroprotective action in Alzheimer's disease, tardive dyskinesia, major depression, epilepsy, and other related neurodegenerative and neuropsychiatric disorders. The mechanism of its neuroprotective action is not completely understood. However, it has been hypothesized to act majorly through its anti-inflammatory and antioxidant properties. Also, it is a potent inhibitor of reactive astrocyte expression and thus prevents cell death. Curcumin also modulates various neurotransmitter levels in the brain. The present review attempts to discuss some of the potential protective role of curcumin in animal models of major depression, tardive dyskinesia and diabetic neuropathy. These studies call for well planned clinical studies on curcumin for its potential use in neurological disorders.

Curcumin is the main biological active phytochemical component of turmeric which is a member of the *Curcuma* botanical group (Family Zingiberaceae). Extensive studies within the last half a century have demonstrated the protective action of curcumin in almost all the disorders of the body. The molecule is known to possess antimicrobial, antiinflammatory, antihypertensive, antihyperlipidemic, antitumor, anticancer, antiphlogistic, antidiabetic, antipsoriasis, antithrombotic, antihepatotoxic and many other useful properties. Besides its protective action in peripheral organ disorders, the molecule is known to possess neuroprotective properties as well (fig. 1)[1]. The low molecular weight and polar structure of curcumin allows it to penetrate the blood-brain barrier effectively. Animal studies have indicated that curcumin can enhance the adult hippocampus neurogenesis process by increasing the number of newly generated cells in the dentate gyrus region of hippocampus[2]. Moreover, it is a potent inhibitor of reactive astrocyte expression and thus, prevents hippocampal cell death induced by kainic acid[3]. In one of the recent studies, low doses of curcumin has shown to effectively disaggregate beta amyloid as well as prevents fibril and oligomer formation and thus found to be protective in treating Alzheimer's disease[1]. Various experimental evidences have shown protective effect of curcumin in animal models of seizures. The molecule is active against amygdaloid kindled seizures in rats[4], iron-induced experimental model of epileptogenesis[5] and electroshock seizures in mice[6]. Similarly, antidepressant activity of curcumin has been reported in animal models of depression. Recently, research on exploring antidepressant properties of curcumin is exponentially increasing (fig. 2). The molecule is effective in forced swim test and chronic unpredictable stress[7,8]. Curcumin possess antidepressant activity through modulating the release of serotonin and dopamine. Curcumin enhances the level of neurotrophic factors such as brain derived neurotrophic factor (BDNF)[9]. Another exciting use of curcumin is in the treatment of diabetic neuropathy. Curcumin enhanced the glucose lowering effect of insulin and protects against the onset of diabetic neuropathy[10]. Our earlier experience with curcumin has demonstrated its neuroprotective action in animal models of tardive dyskinesia. Although, curcumin has shown protective action in many disorders of the body, however, its use is limited due to poor oral bioavailability. However, we have demonstrated in our studies that bioavailability of curcumin can be enhanced by combining it with some bioavailability enhancing agents such as piperine[11].

**Fig. 1:**
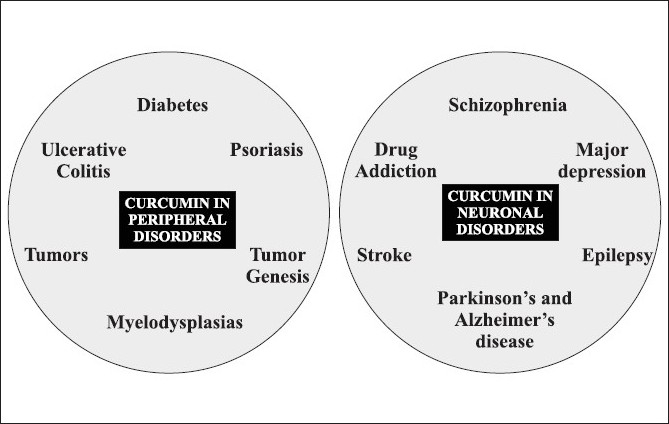
Therapeutic effects of curcumin in several human disorders

**Fig. 2:**
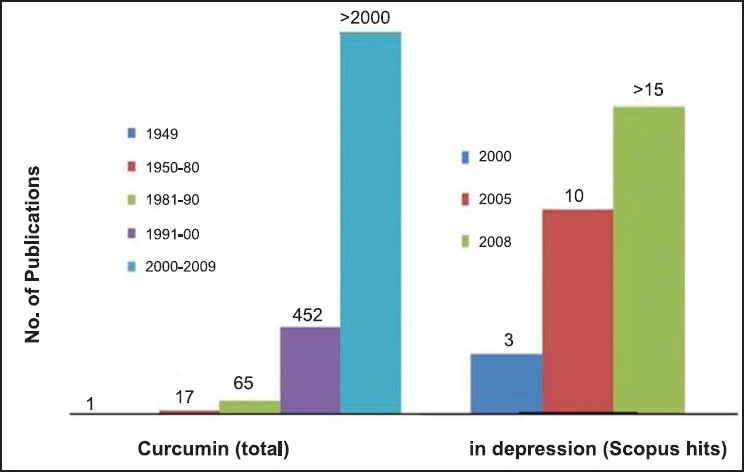
Scopus hits using curcumin and depression as keywords. Source: www.scopus.com

With all this background, the present review attempts to describe the effect of curcumin in animal models of major depression, tardive dyskinesia and diabetic neuropathy. Further, the mechanisms behind the protective action of curcumin in these disorders have also been illustrated.

## Curcumin in major depression:

Major depression is a severe neurological disorder characterized by depressed or irritable mood, decreased interest in pleasurable activities, significant weight loss or gain, insomnia or hypersomnia, psychomotor agitation or retardation, fatigue or loss of energy, feeling of worthlessness or excessive guilt, decreased concentrating power and increase in suicidal tendencies. Approximately, 15-20% of the world population suffers from this disorder at any particular time. Despite the availability of various antidepressants, we are still not able to treat 20-30% of the depressed patients. Also, these antidepressants are associated with plethora of side-effects and drug-drug/drug-food interactions. Therefore, it is utmost important to find alternative drug therapies that is efficacious and safe in the treatment of major depression.

Curcumin has been found to possess antidepressant action in various animal models of depression. Curcumin at dose range of 10-80 mg/kg, i.p. demonstrated antiimmobility action in forced swim test during 6 min period. The maximum anti-immobility effect was observed at 90 min of its administrations[8]. Moreover, curcumin at doses of 40 and 80 mg/kg also reversed the reserpine-induced behavioral despair in mice[8]. Further studies have demonstrated that curcumin enhanced the anti-immobility effect of tranylcypromine (5 mg/kg, i.p.), and selegiline (5 mg/kg, i.p.), two monoamine oxidase (MAO) inhibitors in mouse forced swim test[8]. This study suggests the involvement of monoamine oxidase enzyme in the antidepressant property of curcumin. It was further explored that curcumin inhibits the activity of both MAO-A and MAO-B enzymes. It is important to mention here that monoamine oxidase is the enzyme that is involved in the degradation of norepinephrine, serotonin and dopamine. By inhibiting the activity of MAO enzyme, curcumin increases the concentration of these neurotransmitters in the synapse and thus prolonging their action.

The antidepressant activity of curcumin was further explored by combining it with various conventional and newly discovered antidepressants. To this end, we found that curcumin enhanced the anti-immobility effect of sub-effective doses of f1uoxetine (selective serotonin reuptake inhibitor), bupropion (dopamine reuptake inhibitor) and venlafaxine (dual reuptake inhibitor of serotonin and norepinephrine)[8]. Interestingly, curcumin did not potentiate the antidepressant effect of desipramine (tricyclic antidepressant and norepinephrine reuptake inhibitor) and imipramine (tricyclic antidepressant)[8]. This gives an indication of the involvement of serotoninergic and dopaminergic system in the antidepressant action of curcumin. When the brain neurotransmitter levels were checked following curcumin administration, it increased the levels of serotonin and dopamine but not norepinephrine in the mouse brain[8]. Further, curcumin potentiated the brain levels of serotonin when combined with various antidepressant agents[8]. Therefore, from all the above findings, it can be concluded that the antidepressant action of curcumin majorly involves the serotoninergic neurotransmission. This is further confirmed from the study by Wang and colleagues, who have demonstrated that curcumin antidepressant action is blocked by *p*-chlorophenylalanine, a tryptophan hydroxylase inhibition[9]. Moreover, it has been demonstrated that the antidepressant action of curcumin involves the participation of 5-HT(1A/1B) and 5-HT(2C) receptors[9]. However, in contrast to these studies, Xu and colleagues have demonstrated an increase in noradrenaline levels in the frontal cortex and striatum regions of the rat brain following curcumin administration[12].

Another animal model of depression is unpredictable chronic stress. Our laboratory has demonstrated the protective action of curcumin in unpredictable chronic stress model. In brief, animals were subjected to stress paradigm once a day over a period of 21 days. Some of the unpredictable stressors include, cold swimming (8° for 5 min); tail pinch (1 min); food and water deprivation (24 h); swimming at room temperature (24±2°, 20 min); overnight illumination; no stress; tail pinch (1.5 min); cold swim (l0° for 5 min); swimming at room temperature (24±2° for 15 min); tail pinch (2 min); cold swim (6° for 5 min)[7]. It was found that rats who were chronically challenged to various stressful conditions exhibited significant increase in immobility period as compared to control animals in forced swim test. Curcumin at doses of 20 and 40 mg/kg reversed this immobility period in unpredictable stressed mice[7]. Animal challenged with chronic unpredictable stress demonstrates lower levels of norepinephrine, serotonin and dopamine in the brain. Chronic administration of curcumin did not affect depleted norepinephrine levels but restored levels of serotonin and dopamine[7]. In one of the studies carried out by Li and colleagues, curcumin produced beneficial effects on the stressed rats by effectively improving chronic unpredictable mild stress -induced low sucrose consumption and reducing serum corticosterone levels in rats. The studies further demonstrate the participation of adenylyl cyclase (AC) and cyclic adenosine monophosphate (cAMP) pathway in the antidepressant activity of curcumin[13].

**Fig. 3:**
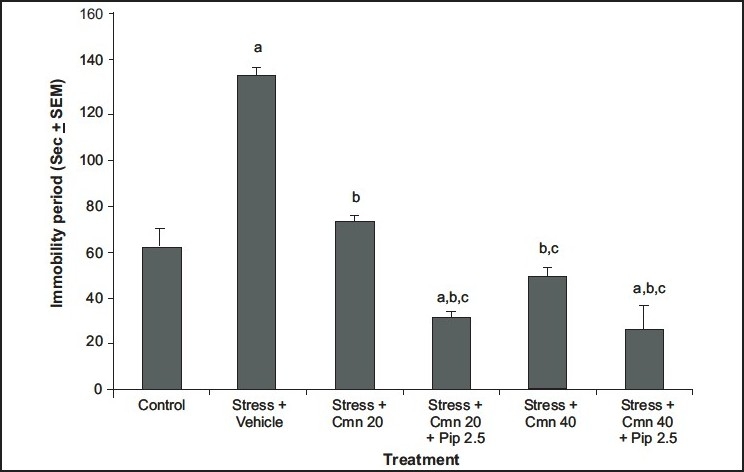
Effect of curcumin alone and along with piperine on forced swim-induced immobility period in rats Effect of curcumin (Cmn) and its combination with piperine (Pip) on forced swim-induced immobility period in rats. ^a^p≤0.05 as compared with control group; ^b^p≤0.05 as compared with stress (S)+vehicle group. ^c^p≤0.05 as compared with stress (S)+curcumin (20). Reproduced with permission from[7].

**Fig. 4:**
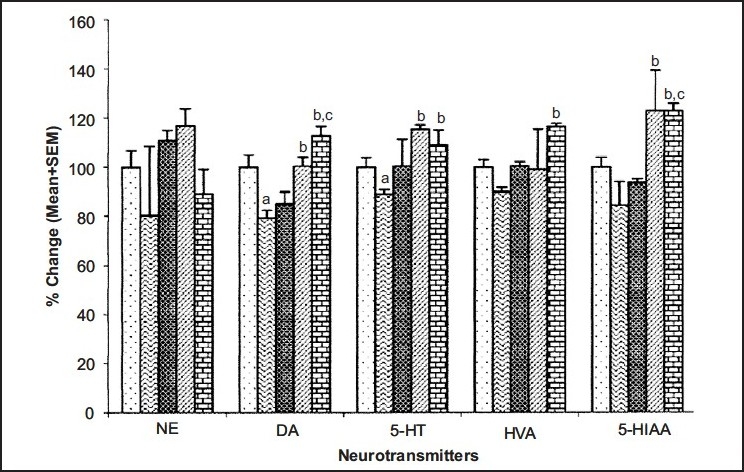
Effect of curcumin and its combination with piperine on brain monoamine levels Effect of curcumin (Cmn) and its combination with piperine (Pip) on brain monoamine levels. ^a^p≤0.05 as compared with control group. ^b^p≤0.05 as compared with stress (S)+vehicle group, ^c^p≤0.05 as compared with stress (S)+curcumin (20). Control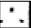, Stress (S)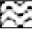, S+curcumin (Cmn) 20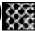, S+Cmn 40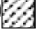, S+Cmn 40+piperine (Pip) 2.5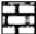. Reproduced with permission from[7].

Curcumin has poor oral bioavailability. In one of the studies carried out in our laboratory, curcumin was administered along with piperine (bioavailability enhancing agent) and animals were challenged to stress paradigm for 21 days followed by measurement of immobility period in forced swim test. It has been demonstrated that piperine (2.5 mg/kg, i.p.) enhanced the antidepressant-like activity of curcumin (20 and 40 mg/kg, i.p.) in mouse challenged to unpredictable mild chronic stress[7] (fig. 3) as compared to vehicle control group. When sub-threshold dose of piperine (2.5 mg/kg, i.p., 21 days) was combined with curcumin (20 mg/kg, i.p., 21 days), there was significant increase in monoamine levels (serotonin, dopamine, homovanillic acid and 5-hydroxyindoleacetic acid) as compared to curcumin (20 mg/kg. i.p., 21 days) *per se* group (fig. 4)[7]. Various mechanisms have been proposed for the antidepressant activity of curcumin. These are tabulated in Table 1.

**TABLE 1:** MECHANISMS PROPOSED FOR ANTIDEPRESSANT ACTIVITY OF CURCUMIN

Monoamine oxidase (MAO) inhibitory property of curcumin
Modulating the serotonin and dopamine neurotransmission in brain
Increasing the levels of neurotrophic factors, particularly brain derived neurotrophic factor (BDNF)
Antiinflammatory and antioxidant property

## Curcumin in Tardive dyskinesia:

Tardive dyskinesia (TD) is a motor disorder of the orofacial region resulting from chronic neuroleptic treatment[11]. TD is characterized by repetitive involuntary movement in the orofacial regions and sometimes limb and trunk musculature. Features of the disorder may include grimacing, tongue protrusion, lip smacking, and rapid eye blinking. Rapid movements of the arms, legs, and trunk may also occur. Involuntary movements of the fingers may appear as though the patient is playing an invisible guitar or piano. There is no standardized treatment available for the treatment of TD. The first step is generally to stop or minimize the use of the neuroleptic drug. However, if the patients have severe symptoms of schizophrenia, than neuroleptics are often replaced by other drugs such as benzodiazepines, adrenergic antagonists, and dopamine agonists.

**Fig. 5:**
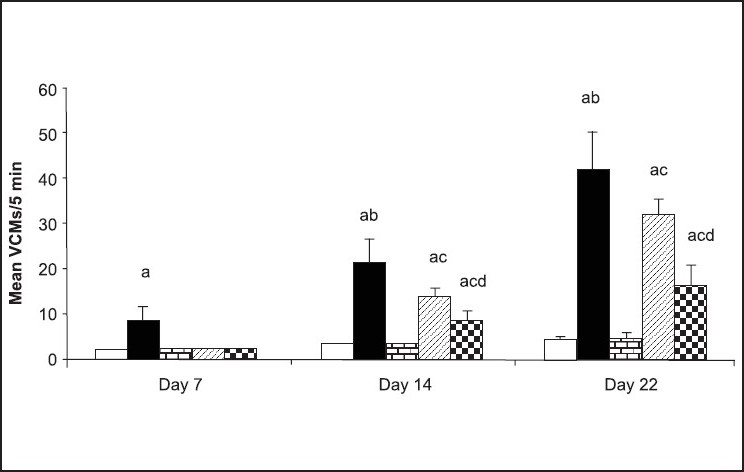
Vacuous chewing movements recorded on days 7, 14 and 22 in chronically-treated rats Vacuous chewing movements (VCM's) recorded on days 7, 14, 22 (test day) in rats chronically treated with (a) vehicle (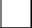), haloperidol (1) mg/kg. i.p. 21 days,(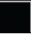), curcumin (50)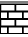, curcumin (25)+haloperidol (1) (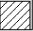). curcumin (50)+haloperidol (l) (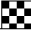). Total number of animals in each group is 5-6 and data is expressed in Mean SEM. ^a^p≤0.05 as compared to control group (on the day of behavioural assessment), ^b^p≤0.05 as compared to haloperidol-treated group of previous week, ^c^p≤0.05 as compared to haloperidol-treated group (on the day of behavioral assessment), ^d^p≤0.05 as compared to Curcumin (25)+ha1operidol-treated group Reproduced with permission from[14].

**Fig. 6:**
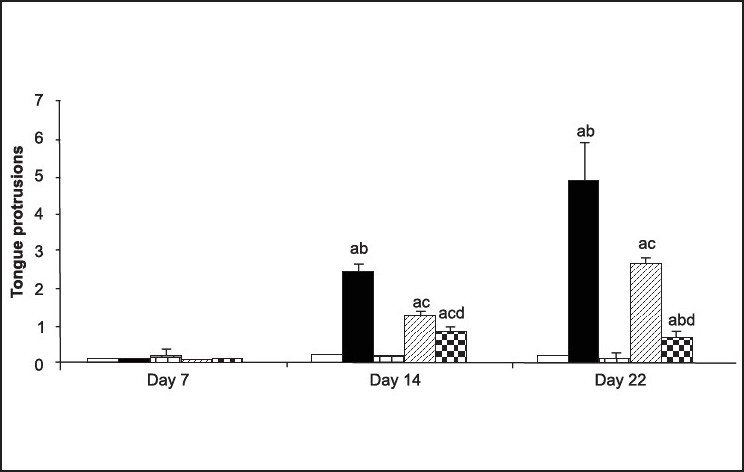
Tongue protrusions recorded on day 7, 14 and 22 in chronicallytreated rats Tongue protrusions recorded on day 7, 14.22 (test day) in rats chronically treated with (a) vehicle (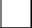), haloperidol (1 mg/kg. i.p. 21 days,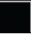), curcumin (50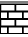), curcumin (25)+haloperidol (1) (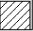). curcumin (50)+haloperidol (l) (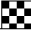). Total number of animals in each group is 5-6 and data is expressed in Mean±SEM. ^a^p≤0.05 as compared to control group (on the day of behavioural assessment), ^b^p≤0.05 as compared to haloperidol-treated group of previous week, ^c^p≤0.05 as compared to haloperidol-treated group (on the day of behavioral assessment), ^d^p≤0.05 as compared to Curcumin (25)+ha1operidoltreated group Reproduced with permission from[11].

However, all the above mentioned drugs have been associated with their own side-effects. Therefore, keeping this in mind, there is need for some of the other alternative treatments for management of TD. In our laboratory, we have explored the effect of curcumin in animal models of TD. Orofacial dyskinesia was induced in rats by administering haloperidol (1 mg/kg, i.p.) chronically to rats for a period of 21 days. After 24 h of last dose of haloperidol injection, vacuous chewing movements (VCMs) were counted. These VCMs were referred to as single mouth openings in the vertical plane not directed toward physical material. If tongue protrusion or VCMs occurred during a period of grooming, they were not taken into account[11]. The VCMs and tongue protrusion was calculated for total of 5 minutes. It has been found that chronic administration of haloperidol (1 mg/kg, i.p.) for a total of 21 days resulted into significant increase in VCM's, tongue protrusion and facial jerking. Interestingly, chronic treatment with curcumin (25 and 50 mg/kg) dose-dependently inhibited the increase of haloperidol-induced VCMs (fig. 5), tongue protrusions (fig. 6) and facial jerking[11]. Moreover, chronic administration of haloperidol resulted in increased lipid peroxidation, decreased reduced glutathione, superoxide dismutase and catalase levels in different regions of brain which was reversed by pretreatment with curcumin. Neurochemical evidences have demonstrated that haloperidol treatment resulted into decreased levels of dopamine, norepinephrine and serotonin in homogenates of cortical and subcortical regions (including striatum) which was prevented by pretreatment with curcumin (25 and 50 mg/kg)[11]. Based on the above experimental evidences, it can be hypothesized that curcumin can be useful drug therapy for the treatment of TD. It would be interesting to note in clinical trials whether reducing the dose of neuroleptic along with adding curcumin in the drug regimen of patients would improve the symptoms of TD. Various mechanisms have been proposed for the protective effect of curcumin in TD, which are tabulated in Table 2.

**TABLE 2:** MECHANISMS PROPOSED FOR ANTITARDIVE DYSKINESIA ACTIVITY OF CURCUMIN

Antiinflammatory properties of curcumin
Modulating the norepinephrine, serotonin and dopamine neurotransmission in brain
Modulating different antioxidant defense system in the body

## Curcumin in diabetic neuropathy:

Diabetic neuropathic pain, the common complication of diabetes, is one of the most difficult types of pain to treat[14]. Approximately 50% of people with diabetes will eventually develop nerve damage in some stage of life. This pain arises due to damaging nerves as a result of high blood sugar levels (hyperglycemia). The symptoms include deep pain, most commonly in the feet and legs, loss of the sense of warm or cold accompanied by muscular cramps, numbness, tingling or burning sensation in the extremities, particularly the feet and weakness. Hyperalgesia is the typical characteristic feature of neuropathic pain. There are many drugs available to treat this symptom. These include antidepressants (amitriptyline, doxepin, or duloxetine), antiseizure medications (gabapentin, pregabalin, carbamazepine, and valproate), antiinflammatory, opioids and capsaicin. However, tight control of blood sugar (glucose) is important to prevent symptoms and problems from getting worse. A lack of the understanding of its aetiology, inadequate relief, development of tolerance and potential toxicity of classical antinociceptives warrant the investigation of the newer agents to relieve this pain. The fact that curcumin possessed antiinflammatory properties which included its inhibitory effect on the production of interleukin-8 (IL-8), interleukin-1β (IL-1β) and TNF-α levels prompted us to observe its protective action in animal models of diabetic neuropathy. To this end, we induced diabetes in mice by injecting 200 mg/kg streptozotocin prepared in citrate buffer (pH 4.4, 0.1M) intraperitoneally. Starting from 4^th^ week till 8^th^ week of streptozotocin injection, curcumin was injected in one group of animals. At the end of the 4^th^ week, diabetic animals exhibited decrease in pain threshold from noxious stimuli as compared to control rats. When evaluated in tail-immersion and hot-plate assay, daily administration of curcumin (15-60 mg/kg) for continuous 4 weeks (starting from 4^th^ week) significantly increased the pain threshold from 4^th^ to 8^th^ week compared to control diabetic mice. Further, chronic administration of curcumin reversed an increase in TNF-alpha levels in diabetic rats. Moreover, curcumin also reversed an increase in nitrite concentration induced due to streptozotocin. In another study, curcumin in association with insulin significantly attenuated thermal hyperalgesia and the hot-plate latencies[10]. Based on the above evidences, it can be concluded that curcumin is a novel antinociceptive agent and can be used as a therapeutic option in the treatment of neuropathic pain associated with diabetes mellitus. Various mechanisms have been proposed for the protective effect of curcumin in diabetic neuropathy. These are tabulated in Table 3.

**TABLE 3:** MECHANISMS PROPOSED FOR PROTECTIVE EFFECT OF CURCUMIN IN DIABETIC NEUROPATHY

Inhibiting the production of TNF-alpha and nitric oxide
Decreasing the nitrite levels in the brain
Inhibiting the production of interleukin- 8 (IL-8), interleukin-lβ (IL-Iβ)

## CONCLUSION

Based on above evidences, it can be concluded that curcumin possessed multiple actions in brain. Curcumin can be a future drug of therapy for the treatment of various neurological disorders such as major depression, tardive dyskinesia and diabetic neuropathy. However, clinical studies are warranted before this molecule is put in use for the therapy.
